# Construction and enhancement of a prototype for a medical-hospital equipment for hypodermoclysis: a qualitative study

**DOI:** 10.1590/0034-7167-2024-0059

**Published:** 2024-09-20

**Authors:** Thayna Silva de Assis Barros, Eduardo José Ferreira Santos, Pedro Miguel dos Santos Dinis Parreira, Juliana Faria Campos, Inês Franco de Almeida, Rafael Alves Bernardes, Marcelle Miranda da Silva

**Affiliations:** I Universidade Federal do Rio de Janeiro. Rio de Janeiro, Rio de Janeiro, Brazil; II Escola Superior de Enfermagem de Coimbra, Unidade de Investigação em Ciências da Saúde: Enfermagem. Coimbra, Portugal

**Keywords:** User-Centered Design, Infusions, Subcutaneous, Equipment and Supplies, Hypodermoclysis, Technology., Diseño Centrado en el Usuario, Infusiones Subcutáneas, Equipos y Suministros, Hipodermoclisis, Tecnología.

## Abstract

**Objectives:**

to construct a Subcutaneous Hydration Device semi-functional prototype and gather initial information to improve this prototype design and realize its acceptance potential.

**Methods:**

a qualitative, descriptive and exploratory study, which used focus group, following the Technology Acceptance Model. The group was held at the Escola Superior de Enfermagem de Coimbra, Portugal, in December 2022, composed of nine participants from six different disciplinary areas, and followed thematic analysis.

**Results:**

four topics emerged associated with the device components: elastomeric infusion pump; needle/access device; clamp; administration set. From these topics, topics were triggered that highlighted: characteristics about the target population; ease of use and accessories; patient comfort and safety; and device application context.

**Final Considerations:**

the Subcutaneous Hydration Device semi-functional prototype is viable and interesting for the clinic. The results support its improvement and direct future investments for experimental studies.

## RESUMEN

**Objetivos:**

construir un prototipo semifuncional del Dispositivo de Hidratación Subcutánea y recopilar información inicial para mejorar el diseño de este prototipo y aprovechar su potencial de aceptación.

**Métodos:**

estudio cualitativo, descriptivo y exploratorio, que utilizó un grupo focal, siguiendo el *Technology Acceptance Model*. El grupo se llevó a cabo en la *Escola Superior de Enfermagem de Coimbra*, Portugal, en diciembre de 2022, estuvo compuesto por nueve participantes de seis áreas disciplinarias diferentes y siguió un análisis temático.

**Resultados:**

surgieron cuatro temas asociados a los componentes del dispositivo: bomba de infusión elastomérica; aguja/dispositivo de acceso; dispositivo de apertura/cierre (clamp); tuberías/equipos. A partir de estos temas se desencadenaron tópicos que resaltaron: características de la población objetivo; facilidad de uso y accesorios; comodidad y seguridad del paciente; y contexto de aplicación del dispositivo.

**Consideraciones Finales:**

el prototipo semifuncional del Dispositivo de Hidratación Subcutánea es viable e interesante para la clínica. Los resultados apoyan su mejora y dirigen futuras inversiones para estudios experimentales.

## INTRODUCTION

Dehydration is defined as a lack of body water due to insufficient intake or excessive losses, or a combination of both^(1)^. Administration of fluids into the subcutaneous tissue is an option when oral rehydration is unsatisfactory or unfeasible^(2)^. In this case, subcutaneous infusion, known as subcutaneous therapy (ST), is an alternative to administering fluids into the subcutaneous tissue that will later be absorbed into the bloodstream^(2-3)^.

ST is reported in the literature as an easy, safe and effective method of parenteral hydration for people at risk of dehydration or with mild to moderate dehydration^(2,4)^, and can be instituted in children, adults and older adults, in varied contexts, such as in a person’s home, in healthcare institutions and in adverse environments such as disasters and wars.

However, despite this versatility and other favorable aspects, such as being considered comfortable and less painful for a person, and easy to insert, with a lower risk of serious complications^(4)^, ST is still underused in clinical practice^(2,4)^.

When used to hydrate, known as hypodermoclysis, the standard technique uses an under-needle device for vascular access adapted for subcutaneous access, a microdroplet administration set device, with or without flow control, which depends on gravitational force, or a perfusion pump, which depends on the electricity or battery^(5)^. The system is assembled by fitting these materials together, which can be detached at any time, which, for use at home, for instance, we believe to be a risk factor for contamination, loss of content or air entering the circuit.

Thus, an innovative device for subcutaneous hydration in humans, Subcutaneous Hydration Device (SHD), was developed with the intention of contributing to its clinical application with quality, agility and safety. This is an incremental innovation, designed by undergraduate students from a nursing school in Coimbra, Portugal, in the Integration to Professional Life discipline, in the innovation module. SHD is registered with the Brazilian National Institute of Industrial Property (INPI - *Instituto Nacional da Propriedade Industrial*) as a utility model^(6)^.

The procedure of joining existing devices and improving their functionalities for hydrating human beings via the subcutaneous route makes SHD an easy-to-use and simplified option, to be presented in the form of a kit for subcutaneous hydration. SHD can improve time and material resource management and benefit home care logistics, because its description informs that its handling can be carried out by people without medical or specialized nursing qualifications^(6)^, making it possible to expand its use in uncontrolled environments.

According to the legal requirements of the Portugal National Authority for Medicines and Health Products (INFARMED - *Autoridade Nacional do Medicamento e Produtos de Saúde*) and European directives, SHD was classified as a class III device (short-term invasive device)^(7)^, being considered a medical device because it refers to a material intended to be used in disease prevention, treatment or relief^(7)^, in this specific case, mild to moderate dehydration, with up to 10% fluid loss^(1-2)^.

## OBJECTIVES

To construct a SHD semi-functional prototype and gather initial information to improve the design of that prototype and realize its acceptance potential.

## METHODS

### Ethical aspects

The study was approved by a Research Ethics Committee. Participants were informed about the study objectives and that participation was voluntary. After reading and signing the Informed Consent Form (ICF), all participants gave their written informed consent. Each participant was identified with a “P”, followed by a number to differentiate them, such as “P1”, and ensure anonymity. Data storage followed all ethical guidelines, in accordance with the General Data Protection Regulation - Law 58/2019, in Portugal.

### Study design

This is a qualitative, descriptive, and exploratory study, which covered the initial objectives of a project that involves assessing SHD usability and conducting a subsequent experimental study. To gather aspects that guide SHD acceptance perception, such as perceived usefulness, ease of use, and attitudes towards use, we applied the Technology Acceptance Model (TAM)^(8-9)^. This report was guided by the COnsolidated criteria for REporting Qualitative research (COREQ) guidelines.

### Procedures

We held a focus group. To meet the standard number of participants in a focus group, i.e., from six to 12 people^(10)^, 15 professionals were recruited, two of whom did not confirm their presence, and four did not attend due to unforeseen circumstances. Thus, the focus group was composed of nine participants.

Participants were recruited using snowball sampling, a form of non-probabilistic sampling used in research where initial participants in a study are encouraged to nominate new participants who, in turn, nominate new participants, and so on, until the required quantity is reached^(11)^.

The 15 potential participants were professionals who had previous contact with other research projects at the institution or were researchers. The invitation was made via telephone call and in person, briefly explaining the study objectives, and was formalized via email after confirming interest in participating.

The focus group session was held in person in a reserved space free from noise and interruptions in December 2022. In addition to the nine participants, seven other research team members were present, with only two acting as moderators. The main researcher, a nurse and one of the moderators, was in the first year of an academic master’s degree in nursing and underwent a training activity to apply the technique. The second moderator was a nurse and professor with extensive experience in this type of investigation.

We applied a questionnaire to characterize the profile of participants (with questions about age, gender, area of professional training and graduate education) and a script to conduct the focus group, such as: what are the strengths you identified in the prototype? In what aspects can the prototype be improved?

On the day of the focus group, the activity was organized as follows: participants arrived one after the other, and, after greeting them, the moderators presented the SHD prototype individually, allowing it to be handled. On this occasion, the sociodemographic questionnaire and the ICF were delivered. In total, the group reception lasted 30 minutes. And then, the focus group began. Everyone introduced themselves to each other, and one of the moderators made a brief introduction about the investigation and its objectives, and explained the session dynamics, which lasted ten minutes. Throughout the session, moderators asked the planned questions, allowing them to be discussed by the group, and asked more questions when necessary, totaling 50 minutes of discussion, ending when no new questions were raised on the topic.

As strategies to carefully analyze and synthesize the discussion, field notes were taken during the focus group and audios were recorded.

### Study setting

The study took place at the *Escola Superior de Enfermagem de Coimbra* (ESEnfC), Portugal, in a meeting room, within the innovation laboratory. This is a partnership between a Brazilian public university, in Rio de Janeiro, and ESEnfC, holder of the utility model, which received the main researcher for the sandwich master’s degree.

### Study participants; inclusion and exclusion criteria

Considering this is the first SHD semi-functional prototype, we initially valued the technical approach, recruiting professionals interested in the field of health technologies. In this case, the heterogeneous focus group is considered more effective due to differences in skills, understanding and knowledge, being essential to highlight diverse information^(12)^. Therefore, we applied professional education at a higher level in health and related areas as the only inclusion criterion. Exclusion criteria covered professionals who had previous contact with the product to be studied.

### Data analysis

The recording was transcribed in full by two researchers independently, following the same criteria, and then the transcription accuracy was checked by the research group. The content was presented to participants for validity, and there were no changes. We used content analysis in the thematic approach, taking into account the assumptions presented by Bardin^(13)^, following the stages of pre-analysis, material exploration, and treatment of results obtained, inference and interpretation. We used WebQDA^®^ software.

The analyzes were complemented with the process flowchart management tool, prepared in Microsoft PowerPoint^®^, to graphically describe, through simple symbols, lines and words, the sequence of the decision process to improve the first SHD semi-functional prototype.

## RESULTS

**Figure 1:**
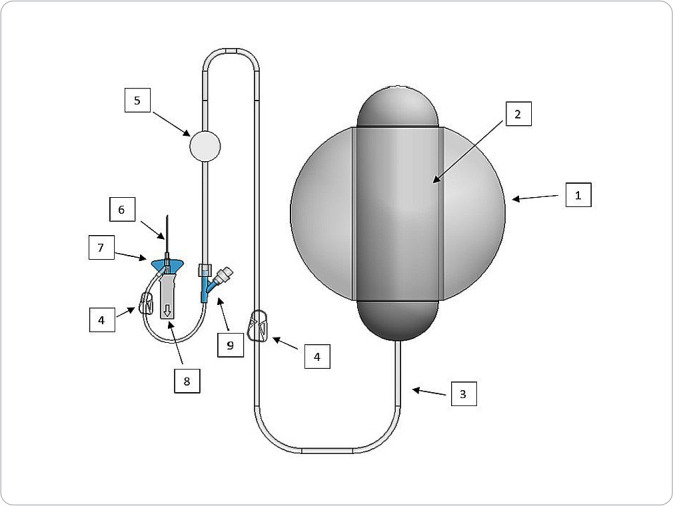
Illustration of the semi-functional Subcutaneous Hydration Device prototype

To structure the SHD semi-functional prototype, commercially available devices were used, selected according to the specifications of the SHD utility model. The volume and infusion flow chosen were the largest available on the market, and as they were determined by the manufacturer, it was not possible to change them. As a result, the prototype was composed of a 400 ml elastomeric infusion pump with an infusion flow of 10 ml/h (1) and a reservoir inside (2). The administration set (3) contains two clamps (4), a particle and air filter (5) and two lumens (9), where the connection was made. The access device contains the catheter (6), flexible and ergonomic wings (7) and a safety device (8) for removing the needle (Figure 1).

As it was a manual and improvised prototyping process, we did not remove components that were not part of the original design, such as the extra clamp and lumen (4 and 9). Participants were instructed to disregard the access device’s second lumen to ensure the idea of a closed system.

This semi-functional prototype (Figure 1) adheres to the SHD utility model concepts, such as a closed system with no possibility of reopening after starting infusion, and the practicality of an elastomeric pump for this type of procedure, which does not require gravity action or electricity.

In relation to the sociodemographic data of the nine focus group participants, the median age was 30 years old and five participants were female. The predominant area of training was nursing, with three participants, followed by pharmaceutical biotechnology, with two participants, and biomedical engineering, biomedical pharmacy, pharmaceutical sciences and biochemistry, with one participant in each. Of the participants, six had a master’s degree and one had a doctoral degree.

The results of the focus group discussion were presented through the main topics based on the components of the SHD semi-functional prototype, namely: elastomeric infusion pump; needle/access device; clamp; and administration set. When discussing each topic, several subtopics were raised, and flowcharts were used to facilitate understanding of the decision process and identify from which point each topic emerged.

### Topic 1: Elastomeric infusion pump - volume and flow

**Figure 2:**
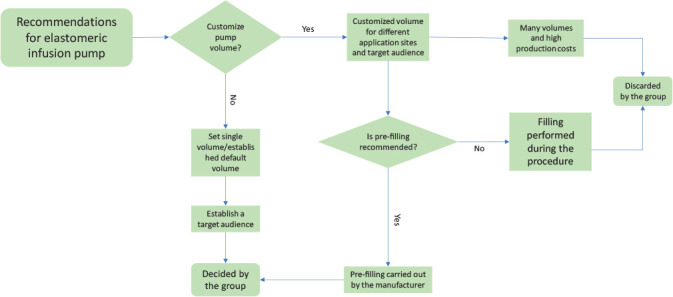
Decision step process for elastomeric infusion pump

The elastomeric pump was presented to participants filled with 400 ml of 0.9% saline. There were several suggestions regarding infusion volume and time, and the variables involved in the decision process, according to the suggestions discussed, are shown in Figure 2, as in the case of the high cost and the need for many units, if volumes were customized, with the group deciding to standardize pump volume and infusion flow.

*I think it might be interesting to try to standardize. Choose an application location* [...] *see how much can be done and make a device for application at that location.* (P3)

When asked about filling the elastomeric pump, participants agreed that it should be pre-filled for product safety and quality.

*Pre-filling is the only way to guarantee non-reuse.* (P5)

*It has to be done in the factory, certifying them* [...] *it already has to come with all the authorizations and all the previous certifications. I think that if the intention is to be a single-use device that is already filled in the place where it is manufactured.* (P4)

The preferred target audience for SHD was discussed as well as possible care environments. Due to its simplified use, the possibilities of greater application of SHD in regions where there are few healthcare professionals were cited as well as for people in home care, as it would be possible for non-healthcare professionals to handle it.

*We had a pandemic, obviously this would help a lot in the hydration process* [...]. *People who are from indigenous lands, people far from the urban center.* (P1)

*I see this a lot in situations where we go to a family’s house and the man or woman is dehydrated and can sleep all night with the pump. In the morning, either the family member or informal caregiver only has to remove it.* (P2)

### Topic 2: Needle/access device

**Figure 3:**
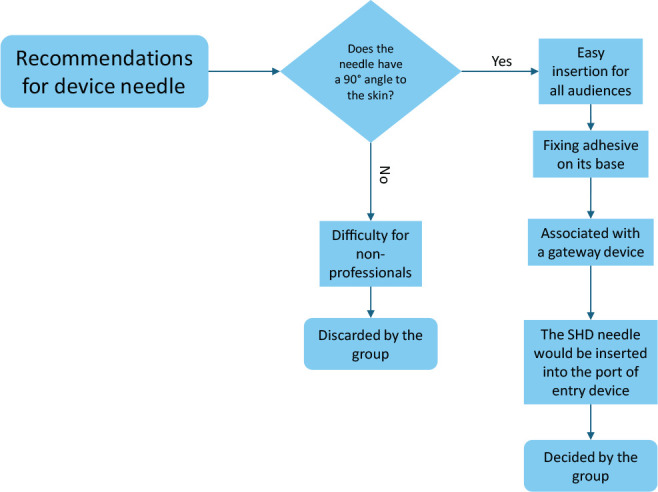
Decision step process for the needle

Regarding the needle that makes up the SHD semi-functional prototype, the topic developed from its angle, as shown in Figure 3. A 90º angle of the needle with the skin was unanimous among participants to guarantee ease of use of SHD.

*If it is not 90º and there are other angles, for a non-technical person, it is a problem, as they will not know what 45º or 30º is.* (P2)

[...] *I think this standardization* (of 90º) *is worth it.* (P3)

For patient comfort and alignment of the device to the skin, it was suggested that the needle be fixed using an adhesive system on the bottom, which is in contact with the skin.

*The needle should be 90º and have an adhesive at its base to fix it on the skin, without the need for an external adhesive.* (P1)

Participants suggested associating the 90º needle with an entry device, similar to existing devices for self-application of insulin, because if patients need to maintain hydration, they will not need to be punctured again.

*There is a device that you can place at any insulin application site and it stays in the subcutaneous tissue for three days, and for you to apply it, you take either an insulin pen or an insulin syringe and apply it through it and it is placed at a 90º angle; it’s like a stamp.* (P2)

*An insulin device that was placed on the user* [patient], *the needle came out and it was stuck for 24 hours or up to 72 hours.* (P1)

*I think this issue of pinch is interesting in palliative care, which involves patient comfort* (P3)

### Topic 3: Clamp

In the semi-functional prototype, there were two clamps, both of which were movable. The first was located at the interior end, and the second, in the administration set. Participants did not consider the presence of two clamps necessary, but this fact supported the initial discussion about where a clamp should be located, with the location close to the access device being chosen at first, mainly to reduce the chances of forgetting to open the system and release the flow.

[...] *just one is enough, we don’t need both* [...] *maybe closer to the tip, like a safety clip that when unlocked it is no longer guaranteed that it can close.* (P4)

*We can leave it at the bottom, with the filter, so that it is only unlocked when inserting.* (P5)

**Figure 4:**
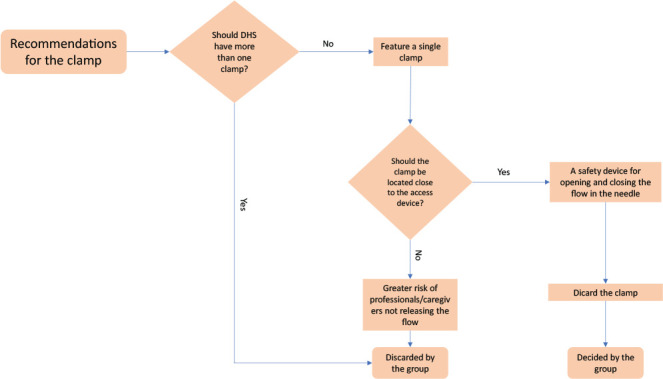
Decision step process for clamp

On the other hand, throughout the discussion, due to the risk of not opening the flow, preventing the saline from being infused, or of closing the flow, unintentionally by a non-professional, in addition to causing pressure injuries related to a medical device, participants suggested adding the functionality of opening and closing the flow in the needle, discarding the use of a clamp in SHD (Figure 4).

*If a caregiver or healthcare professional takes care of a user* [patient], *will they know that they cannot turn lying down on top of the access?* (P1)

*So, the safety system can be in the lower part of the needle, i.e., when unlocked, it automatically infuses.* (P5)

*It had to be on the needle; when pricked, it would remain open. If it is for single use, that is, if the system is broken, it cannot be closed again.* (P7)

*It would have to be an ON/OFF system, or something like that at the end, and we wouldn’t need these clamps.* (P1)

### Topic 4: Administration set

**Figure 5:**
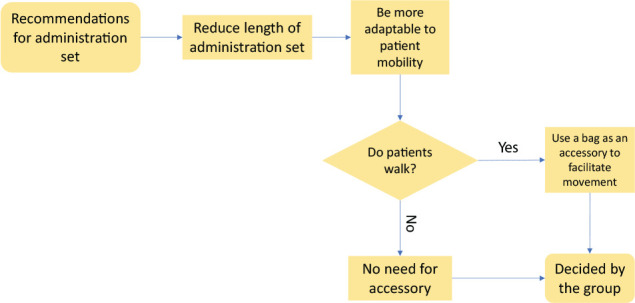
Decision stage process for administration set

The prototype administration set is directly connected to the elastomeric pump, and its end is connected to the access device. It measures approximately 1.20 meters and was considered large by participants (Figure 5).

*My experience with an insulin pump is that it can turn patients around. Sometimes they place the device in the subcutaneous tissue on that side and allows you to loop it around to the other side, or even in your pocket.* (P3)

*I think it’s too big, if it’s to be close to patients. If it goes around the body, ok, but if it doesn’t, there will be a lot of loose thread, and people are likely to tighten it and impede the flow, obstructing it.* (P6)

Lastly, given the opportunity to reach walking patients, it was proposed that the SHD semi-functional prototype had a bag as an accessory to place the elastomeric pump inside it, thus making it easier to carry.

*When we think that if a person is not lying down or doing something, they always have to carry it in their hand, and that is not practical. There are some suitcases that patients can put the device in and make their life normal, and can actually walk.* (P5)

## DISCUSSION

In this study, we constructed the first SHD semi-functional prototype from off-the-shelf devices, and contributions from the heterogeneous focus group were instrumental in gathering initial information to improve the design of this prototype and realize its acceptance potential. The search for the interrelationship between various areas of knowledge to discuss the same object of study resulted in the production of new perspectives on the reality addressed. Therefore, interdisciplinarity in the field of health technologies stands out, as it aligns with the objective of optimizing means for its development^(14-15)^.

Given these circumstances, it is essential to highlight the decision to establish inclusion criteria for the first focus group, recognizing the importance of a technical approach to constructing the prototype. However, from the perspective that its improvement must be progressive, we highlighted the participation of end users in this process as a condition without which success will not be achieved.

It is important to highlight SHD as a facilitator in time management for healthcare professionals, as it eliminates some steps in preparing the materials needed in work environments. The lack of time in healthcare services, or even in patients’ homes, is considered a factor that increases care precariousness and reduces care quality^(16)^.

Strategies that improve time management play an important role. They directly impact clinical results^(17)^, organization and costs, making it possible to expand capacity and access to services, especially those provided in hospitals, as they facilitate transition of care to primary care and to the community, where some procedures and specific care can be carried out, as in the case of hydration^(18)^.

That being said, SHD can be considered a fruitful acquisition, since it has its components in a single device for simplified use, previously filled with solution, presented in the form of a kit, with continuous infusion pre-determined by the manufacturer.

However, it is important to maintain dialogue with economic dimensions, since, when incorporating new health technologies, the value of a product is one of the factors to be analyzed^(19)^. SHD, as it is considered a complete, single-use and pre-filled kit, adds a higher cost of production, transportation and acquisition to both direct consumers and healthcare providers, which may be a limitation of interest to the market.

However, this hypothesis needs to be assessed based on an analytical observation that compares the provision of this same intervention - subcutaneous hydration - in hospital settings, for instance. In this case, including other variables, such as cost of labor, and social factors related to the commuting of patients and, possibly, their companion, can influence these economic results^(18,20)^. Using SHD at home, for instance, can be a preventive measure and capable of reducing the use of emergency services, especially in frail older adults.

In this context, in line with research results, the home environment was highlighted as a possible setting for using SHD. Home care has proven to be a complementary alternative to hospital intervention, aiming to reduce the demand for hospitalizations and reduce patients’ hospitalization period^(18)^. Furthermore, it allows patients to remain in their family context and provides greater applicability of person-centered care, prioritizing personalized and collaborative care^(18,21)^.

Linked to this fact, population aging leads to a greater search for home care. With increased life expectancy, there is an increase in risk factors associated with noncommunicable diseases (NCDs) which, together with aging, generate limitations that can develop permanent or temporary functional disabilities^(21-24)^. Therefore, home care becomes a priority, whether in the prevention of illnesses, treatment, rehabilitation, or palliative care.

Given this reality, the public receiving home care will be SHD’s main target audience. The SHD incorporated to this public becomes attractive to the market and health systems, as this type of care presents lower costs for patient maintenance and better results compared to hospital inpatient services^(18,24)^. Likewise, using SHD at home can reduce professional visits and the costs of their fees.

In home care, it is worth highlighting the importance of a family caregiver, who plays an important role in keeping patients at home^(25)^. They, in turn, may not have the skills to carry out such an activity, which requires health education and the provision of instruments that can facilitate home care^(26)^, as in the case of parenteral hydration. Therefore, the SHD proposal for subcutaneous hydration, simple and easy to use, can allow quick and comfortable handling by people without medical or specialized nursing qualifications, with minimized risks.

Using technologies at home, previously used in hospitals, leads to a greater chance of risk of errors and adverse events^(27)^. In this study, we identified some barriers that caregivers could face when using SHD, such as access device needle angle, as, to puncture, it is necessary to angle it at 45º with the skin^(28)^. Added to the fear of performing a technical gesture, caregivers may not understand the angle to be performed, increasing the risk of errors. Therefore, the angle idealized by the focus group participants was 90º, as it would cause fewer doubts about the subject.

However, this characteristic does not favor application in patients with conditions of intense frailty, such as cachexia and high sarcopenia, due to subcutaneous tissue thickening. In these conditions, protocols indicate using a non-needled catheter and bevel insertion at a 30º angle with the skin^(29)^. Therefore, in practice, it is crucial that healthcare professionals assess patients’ physical condition, but also carry out health education for family members/caregivers, in order to ensure the comfort of both parties when determining the best angle of the SHD needle.

The World Patient Safety Alliance launched the third global challenge with the topic of safe medication, presenting patient and family/caregiver empowerment as one of its objectives^(30)^. Although the initiative focuses on reducing harm related to medication, it is considered that patient and family/caregiver empowerment must occur in all dimensions, complexities and technological densities of care^(31)^.

From this perspective, this study presents points related to autonomy preservation and self-care promotion. Autonomy refers to patients’ participation in decision-making on issues inherent to their care, with respect for dignity^(32)^. In turn, self-care is related to a regulatory function, in which people are allowed to carry out activities aimed at preserving life, health, development and well-being on their own^(33)^.

Considering that autonomy and self-care concepts are complex, the development of technologies that promote patient empowerment is challenging, as they involve ease of use, components adaptable to different environments and characteristics that allow their use. Focusing on these concepts, presenting the SHD semi-functional prototype as a simplified use and easy insertion kit, in addition to associating it with an accessory bag, is a feature that contributes to patient autonomy and self-care.

### Study limitations

The following were limitations: a single focus group, despite it being the first phase of the study; the restriction of higher education as an eligibility criterion; the absence of criteria in characterizing the profile, such as clinical experience and product development, although snowball sampling was aimed at those interested in the field of health technologies; and non-participation of end users (patients and family members). Snowball sampling itself also presented limitations, as it restricted the potential scope and inclusion of the group, since participant selection takes place based on the bond between them, who can share thoughts and opinions. Using devices available on the market also limited prototyping, as in the case of the elastomeric pump volume, but, on the other hand, it made research economically viable.

### Contributions to nursing, health, or public policy

The study is inserted in the context of technology and innovation; therefore, it contributes to the growth of research on the subject in health. It contributes to developing new medical devices, specifically to respond to the needs of patients with mild to moderate dehydration in home environments, facilitating nursing and healthcare management. The study is committed to presenting researchers/healthcare professionals with the importance of prototyping, which can be developed in several stages, due to the need for progressive improvement.

## FINAL CONSIDERATIONS

Innovative medical devices play an important role in managing and promoting health, providing healthcare professionals with strategies to achieve efficient and safe care delivery. However, these devices need to be constantly improved to ensure that their construction and design are in line with their end users and can provide safety for the intended treatment. Therefore, identifying this information about the SHD semi-functional prototype, through the focus group, was positive and capable of identifying potentialities and suggestions for its improvement.

With regard to the focus group, although the results obtained are satisfactory, it is recommended that future studies be carried out to improve the final SHD prototype, with larger and more homogeneous samples to bring more robustness to this process. Furthermore, focusing on SHD usability, studies will be needed that present methodologies involving testing the final prototype in different phases of product development.
