# Supplementary material for: Construction and enhancement of a prototype for a medical-hospital equipment for hypodermoclysis: a qualitative study
Source: Rev Bras Enferm. 2024 Sep 20;77(4):e20240059. doi: 10.1590/0034-7167-2024-0059 (PMC11419691; doi:10.1590/0034-7167-2024-0059)
Supplement: Translation

# Translation: Construção e aprimoramento do protótipo de um equipamento médico-hospitalar para hipodermóclise: estudo qualitativo

**DOI:** 10.1590/0034-7167-2024-0059pt

**Authors:** Thayna Silva de Assis Barros, Eduardo José Ferreira Santos, Pedro Miguel dos Santos Dinis Parreira, Juliana Faria Campos, Inês Franco de Almeida, Rafael Alves Bernardes, Marcelle Miranda da Silva

**Affiliations:** I Universidade Federal do Rio de Janeiro. Rio de Janeiro, Rio de Janeiro, Brasil; II Escola Superior de Enfermagem de Coimbra, Unidade de Investigação em Ciências da Saúde: Enfermagem. Coimbra, Portugal

**Keywords:** *Design* Centrado no Usuário, Infusões Subcutâneas, Equipamentos e Provisões, Hipodermóclise, Tecnologia.

## Abstract

**Objetivos:**

construir protótipo semifuncional do Dispositivo de Hidratação Subcutânea e reunir informações iniciais para aprimorar o *design* desse protótipo e perceber seu potencial de aceitação.

**Métodos:**

estudo qualitativo, descritivo e exploratório, que utilizou o grupo focal, seguindo o *Technology Acceptance Model*. O grupo foi realizado na Escola Superior de Enfermagem de Coimbra, Portugal, em dezembro de 2022, composto por nove participantes de seis áreas disciplinares distintas, e seguiu a análise temática.

**Resultados:**

emergiram quatro temas associados aos componentes do dispositivo: bomba de infusão elastomérica; agulha/dispositivo de acesso; dispositivo de abrir/fechar (*clamp*); tubuladura/equipamento. A partir desses temas, foram desencadeados tópicos que destacaram: características acerca da população-alvo; facilidade de uso e acessórios; conforto e segurança do paciente; e contexto de aplicação do dispositivo.

**Considerações Finais:**

o protótipo semifuncional do Dispositivo de Hidratação Subcutânea é viável e interessante para a clínica. Os resultados subsidiam seu aprimoramento e direcionam futuros investimentos para estudo experimental.

## INTRODUÇÃO

A desidratação é definida pela falta de água corporal devido à ingestão insuficiente ou perdas excessivas, ou uma combinação de ambos^(1)^. A administração de líquidos no tecido subcutâneo é uma opção quando a reidratação por via oral é insatisfatória ou inviável^(2)^. Nesse caso, a infusão subcutânea, conhecida como terapia subcutânea (TS), é uma alternativa para administrar fluidos no tecido subcutâneo que, posteriormente, serão absorvidos na corrente sanguínea^(2-3)^.

A TS está relatada na literatura como um método fácil, seguro e eficaz de hidratação parenteral para pessoas com risco de desidratação ou com desidratação leve a moderada^(2,4)^, podendo ser instituída em crianças, adultos e idosos, em contextos variados, como no domicílio da pessoa, nas instituições de saúde e em ambientes adversos de situações de desastres e guerras.

Entretanto, apesar dessa versatilidade e de outros aspectos favoráveis, como ser considerada confortável e menos dolorosa para a pessoa, e de fácil inserção, com menor risco de complicações graves^(4)^, a TS ainda é subutilizada na prática clínica^(2,4)^.

Quando utilizada para hidratar, nomeada como hipodermóclise, a técnica padrão utiliza um dispositivo sob agulha para acesso vascular adaptado para o acesso subcutâneo, equipo microgotas com ou sem controle de fluxo, que depende da força gravitacional, ou bomba de perfusão, que depende da eletricidade ou bateria^(5)^. O sistema é montado por encaixe desses materiais, que podem ser desencaixados a qualquer momento, o que, para uso em domicílio, por exemplo, acreditamos ser um fator de risco de contaminação, de perda de conteúdo ou de entrada de ar no circuito.

Dessa forma, um dispositivo inovador para a hidratação subcutânea em seres humanos, o Dispositivo de Hidratação Subcutânea (DHS), foi desenvolvido com a intenção de contribuir para a sua aplicação clínica com qualidade, agilidade e segurança. Trata-se de uma inovação incremental, idealizada por alunos de graduação de uma escola de enfermagem em Coimbra, Portugal, na disciplina Integração à Vida Profissional, no módulo de inovação. O DHS encontra-se registrado no Instituto Nacional da Propriedade Industrial (INPI) como modelo de utilidade^(6)^.

O procedimento de juntar dispositivos já existentes e melhorar suas funcionalidades para hidratação de seres humanos pela via subcutânea torna o DHS uma opção de fácil uso e simplificada, a ser apresentado na forma de um *kit* para hidratação subcutânea. O DHS pode melhorar a gestão do tempo e dos recursos materiais e beneficiar a logística da assistência domiciliar, porque sua descrição informa que sua manipulação pode ser realizada por pessoas sem habilitações médicas ou de enfermagem especializada^(6)^, sendo possível ampliar seu uso em ambientes não controlados.

Segundo os requisitos legais da Autoridade Nacional do Medicamento e Produtos de Saúde (INFARMED) de Portugal e as diretivas europeias, o DHS foi classificado como dispositivo de classe III (dispositivo invasivo de curta duração)^(7)^, sendo considerado um dispositivo médico porque se refere a um material destinado a ser usado na prevenção, no tratamento ou no alívio de uma doença^(7)^, nesse caso específico, da desidratação leve a moderada, com até 10% de perda de fluidos^(1-2)^.

## OBJETIVOS

Construir um protótipo semifuncional do DHS e reunir informações iniciais para aprimorar o *design* desse protótipo e perceber seu potencial de aceitação.

## MÉTODOS

### Aspectos éticos

O estudo foi aprovado pelo Comitê de Ética em Pesquisa. Os participantes foram informados sobre os objetivos do estudo e que a participação era voluntária. Todos, após a leitura e assinatura do Termo de Consentimento Livre e Esclarecido (TCLE), deram seu consentimento informado por escrito. Cada participante foi identificado com um “P”, seguido de um número para diferenciá-los, por exemplo “P1”, e garantir anonimato. O armazenamento dos dados seguiu todas as orientações éticas, de acordo com a Regulamentação Geral de Proteção de Dados - Lei n.º 58/2019, em Portugal.

### Tipo de estudo

Estudo qualitativo, descritivo e exploratório, que abrangeu os primeiros objetivos de um projeto que envolve a avaliação da usabilidade do DHS e posterior estudo experimental. Para reunir aspectos que orientem a percepção sobre a aceitação do DHS, como utilidade percebida, facilidade de uso e atitudes em relação ao uso, aplicamos o *Technology Acceptance Model* (TAM)^(8-9)^. Esse relatório foi guiado pelas diretrizes do *COnsolidated criteria for REporting Qualitative research* (COREQ).

### Procedimentos

Realizamos um grupo focal. Para atender ao padrão do número de participantes de um grupo focal, ou seja, de seis a 12 pessoas^(10)^, foram recrutados 15 profissionais, sendo que dois não confirmaram presença e quatro não compareceram devido a imprevistos. Dessa forma, o grupo focal foi composto por nove participantes.

O recrutamento dos participantes ocorreu pela técnica *snowball*, uma forma de amostra não probabilística usada em pesquisas onde os participantes iniciais de um estudo são incentivados a indicar novos participantes que, por sua vez, indicam novos participantes, e assim sucessivamente, até que seja alcançado o quantitativo necessário^(11)^.

Os 15 potenciais participantes eram profissionais que tiveram contato anterior com outros projetos de investigação da instituição ou eram pesquisadores. O convite ocorreu por ligação telefônica e presencialmente, explicando brevemente os objetivos do estudo, tendo sido formalizado via e-mail após confirmado o interesse em participar.

A sessão do grupo focal foi realizada presencialmente em espaço reservado e livre de ruídos e interrupções em dezembro de 2022. Além dos nove participantes, estavam presentes outros sete membros da equipe de pesquisa, sendo que apenas dois atuaram como moderadores. A pesquisadora principal, enfermeira e uma das moderadoras, encontrava-se no primeiro ano do curso do mestrado acadêmico em enfermagem, e passou por uma atividade de formação para aplicação da técnica. O segundo moderador era um enfermeiro e docente, com vasta experiência nesse tipo de investigação.

Aplicamos um questionário para caracterização do perfil dos participantes (com questões sobre idade, gênero, área de formação profissional e de pós-graduação) e um roteiro para conduzir o grupo focal como: quais os pontos fortes que identificou no protótipo? Em quais aspectos o protótipo pode ser melhorado?

No dia do grupo focal, a atividade foi organizada da seguinte forma: os participantes foram chegando um após o outro, e, após cumprimentá-los, os moderadores apresentavam o protótipo do DHS individualmente, permitindo o seu manuseio. Nessa oportunidade, eram entregues o questionário sociodemográfico e o TCLE. Ao todo, a recepção do grupo durou 30 minutos. E então, procedeu-se ao início do grupo focal. Todos se apresentaram mutuamente, e um dos moderadores fez uma breve introdução sobre a investigação e seus objetivos, e explicou a dinâmica da sessão, o que durou dez minutos. Ao longo da sessão, os moderadores realizaram as perguntas planejadas, permitindo que fossem discutidas pelo grupo, e indagavam mais perguntas quando necessário, perfazendo 50 minutos de discussão, encerrada quando nenhum novo questionamento foi levantado sobre o tema.

Como estratégias para analisar e resumir cuidadosamente a discussão, foram realizadas notas de campo durante o grupo focal e gravação de áudio.

### Cenário do estudo

O local do estudo foi na Escola Superior de Enfermagem de Coimbra (ESEnfC), Portugal, em sala de reuniões, do laboratório de inovação. Trata-se de uma parceria entre uma universidade pública brasileira, no Rio de Janeiro, e a ESEnfC, titular do modelo de utilidade, que recebeu a pesquisadora principal para o mestrado sanduíche.

### Participantes do estudo; critérios de inclusão e exclusão

Considerando tratar-se do primeiro protótipo semifuncional do DHS, valorizamos, inicialmente, a abordagem técnica, recrutando profissionais interessados pelo campo das tecnologias em saúde. Nesse caso, o grupo focal heterogêneo é considerado mais eficaz devido às diferenças de competências, compreensão e conhecimento, sendo essencial para evidenciar diversas informações^(12)^. Assim, aplicamos como único critério de inclusão a educação profissional em nível superior na área da saúde e correlatas. O critério de exclusão abrangeu os profissionais que tiveram contato prévio com o produto a ser estudado.

### Análise de dados

A gravação foi transcrita na íntegra por duas pesquisadoras de forma independente, seguindo os mesmos critérios, e depois verificada a precisão das transcrições pelo grupo de pesquisa. O conteúdo foi apresentado aos participantes para validação, e não houve alterações. Utilizamos a análise de conteúdo na abordagem temática, tendo em conta os pressupostos apresentados por Bardin^(13)^, seguindo as etapas da pré-análise, exploração do material, e tratamento dos resultados obtidos, inferência e interpretação. Utilizamos o *software* WebQDA^®^.

As análises foram complementadas com a ferramenta de gestão para fluxograma de processos, elaborado no *Microsoft PowerPoint* ^®^, de modo a descrever graficamente, por meio de símbolos simples, linhas e palavras, a sequência do processo de decisão para aprimoramento do primeiro protótipo semifuncional do DHS.

## RESULTADOS

Para estruturar o protótipo semifuncional do DHS, foram utilizados dispositivos disponíveis no mercado, selecionados de acordo com as especificações do modelo de utilidade do DHS. O volume e o fluxo de infusão escolhidos foram os maiores disponíveis no mercado, e por serem determinados pelo fabricante, não foi possível alterá-los. Como resultado, o protótipo foi composto por uma bomba de infusão elastomérica de 400 ml com o fluxo de infusão de 10 ml/h (1) e seu reservatório no interior (2). No equipo (3), estão localizados dois *clamps* (4), um filtro de partículas e ar (5) e dois lúmens (9), onde foi realizada a sua conexão. No dispositivo de acesso, encontram-se o cateter (6), asas flexíveis e ergonômicas (7) e um dispositivo de segurança (8) para a retirada da agulha (Figura 1).

**Figura 1:** Ilustração do protótipo semifuncional do Dispositivo de Hidratação Subcutânea

Por ter se tratado de um processo manual e improvisado de prototipagem, não retiramos os componentes que não faziam parte do *design* original, como o *clamp* e o lúmen extras (4 e 9). Os participantes foram orientados a desconsiderar o segundo lúmen do dispositivo de acesso para garantir a ideia de sistema fechado.

Esse protótipo semifuncional (Figura 1) fideliza os conceitos do modelo de utilidade do DHS, como o sistema fechado, sem possibilidade de reabertura após início da infusão, e a praticidade de uma bomba elastomérica para esse tipo de procedimento, que não requer ação da gravidade ou eletricidade.

Acerca dos dados sociodemográficos dos nove participantes do grupo focal, a mediana das idades foi de 30 anos e cinco participantes eram do sexo feminino. A área de formação predominante foi a enfermagem, com três participantes, seguida da biotecnologia farmacêutica, com dois participantes, e engenharia biomédica, farmácia biomédica, ciências farmacêuticas e bioquímica, com um participante em cada. Dos participantes, seis possuíam mestrado e um possuía doutorado.

Os resultados da discussão do grupo focal foram apresentados por meio dos temas principais baseados nos componentes do protótipo semifuncional do DHS, sendo eles: bomba de infusão elastomérica; agulha/dispositivo de acesso; dispositivo de abrir/fechar; e tubuladura/equipo. Ao discutir cada tema, alguns tópicos foram levantados, e os fluxogramas foram utilizados para facilitar a compreensão do processo de decisão e identificar de qual ponto surgiu cada tópico.

### Tema 1: Bomba de infusão elastomérica - volume e fluxo

A bomba elastomérica foi apresentada aos participantes preenchida com 400 ml de soro fisiológico 0,9%. Foram várias as sugestões em relação ao volume e ao tempo de infusão, e as variáveis implicadas no processo de decisão, de acordo com as sugestões discutidas, estão dispostas na Figura 2, como no caso do alto custo e da necessidade de muitas unidades, se personalizados os volumes, tendo o grupo decidido por padronizar o volume da bomba e o fluxo de infusão.

**Figura 2:** Processo da etapa de decisão para a bomba de infusão elastomérica

*Acho que talvez fosse interessante tentar padronizar. Escolher um local de aplicação* [...] *vê quanto que pode ser feito e faz um dispositivo para aplicação neste local.* (P3)

Ao serem questionados sobre o preenchimento da bomba elastomérica, os participantes entraram em acordo de que ela deveria vir preenchida, para segurança e qualidade do produto.

*O preenchimento prévio é a única maneira de se conseguir garantir a não reutilização.* (P5)

*Tem que ser em fábrica, certificá-las* [...] j*á tem que ir com todas as autorizações e todas as certificações prévias. Eu acho que se a intenção é ser um dispositivo de utilização única que já vá preenchido no local que é fabricado.* (P4)

Discutiu-se o público-alvo preferencial para o DHS, bem como os possíveis ambientes de cuidado. Por ser de uso simplificado, foram citadas as possibilidades de maior aplicação do DHS nas regiões onde existem poucos profissionais de saúde, bem como para pessoas em cuidados domiciliares, pois seria possível o manuseio por não profissionais de saúde.

*Nós tivemos uma pandemia, óbvio que isso ajudaria muito no processo de hidratação* [...]. *Pessoas que são de terra indígenas, pessoas afastadas do centro urbano.* (P1)

*Eu vejo isto muito em situações em que nós vamos à casa de uma família e o senhor ou senhora está desidratado e pode dormir toda a noite com a bomba. De manhã, ou o familiar ou cuidador informal só tem que retirar.* (P2)

### Tema 2: Agulha/dispositivo de acesso

Ao que se refere à agulha que compõe o protótipo semifuncional do DHS, a temática se desenvolveu a partir da sua angulação, conforme Figura 3. A angulação de 90º da agulha com a pele foi unânime entre os participantes, para garantia da facilidade de uso do DHS.

**Figura 3:** Processo da etapa de decisão para a agulha

*Se não for a 90º e forem outros ângulos, para uma pessoa não técnica, é um problema, pois não saberá o que é 45º ou 30º.* (P2)

[...] *eu acho que essa padronização* [de 90º] *vale à pena.* (P3)

Para o conforto do paciente e o alinhamento do dispositivo à pele, foi sugerido que a fixação da agulha fosse realizada por um sistema adesivo na parte inferior, que está em contato com a pele.

*A agulha deveria ser de 90º e ter um adesivo em sua base para fixar na pele, sem precisar de um adesivo externo.* (P1)

Os participantes sugeriram associar a agulha de 90º a um dispositivo de porta de entrada, similar a dispositivos já existentes para autoaplicação de insulina, pois caso o paciente tenha a necessidade de manter a hidratação, não precisará ser puncionado novamente.

*Há um dispositivo que você pode colocar em qualquer local de aplicação de insulina e ele fica três dias no tecido subcutâneo, e para você aplicar, você pega ou uma caneta de insulina ou uma seringa de insulina e aplica através dele e é de colocação de 90º; é como se fosse um carimbo.* (P2)

*Um dispositivo para insulina em que ele colocava no utente* [paciente]*, a agulha saía e ele ficava preso por 24 horas ou até 72 horas.* (P1)

*Eu penso que essa questão das picadas é interessante em cuidados paliativos que envolve o conforto do paciente* (P3)

### Tema 3: Dispositivo de abrir/fechar (*clamp*)

No protótipo semifuncional, havia dois dispositivos de abrir e fechar (*clamp*), sendo os dois móveis. O primeiro estava localizado na extremidade interior, e o segundo, na tubuladura/equipo. Os participantes não consideraram necessária a presença dos dois *clamps*, mas esse fato sustentou a discussão inicial sobre onde o *clamp* deveria ser localizado, sendo escolhido, em um primeiro momento, o local próximo ao dispositivo de acesso, principalmente para diminuir as chances de esquecerem de abrir o sistema e liberar o fluxo.

[...] *basta só um, não precisamos dos dois* [...] *se calhar, mais perto da ponta, como um clip de segurança que ao ser destravado já não se garante que pode se fechar.* (P4)

*Podemos deixar na altura de baixo, com o filtro, que seja desbloqueado só na hora de inserir.* (P5)

Por outro lado, ao longo da discussão, devido ao risco de não abrir o fluxo, impedindo que o soro fisiológico seja infundido, ou de fechar o fluxo, sem intenção por um não profissional, além de causar lesão por pressão relacionada a dispositivo médico, os participantes sugeriram acrescentar a funcionalidade de abrir e fechar o fluxo na agulha, descartando o uso do *clamp* no DHS (Figura 4).

**Figura 4:** Processo da etapa de decisão para o dispositivo de abrir/fechar (*clamp*)

*Se o cuidador ou o profissional de saúde tiver o cuidado com o utente* [paciente]*, ele vai saber que não pode virar de decúbito em cima do acesso?* (P1)

*Então, o sistema de segurança pode ser naquela parte de baixo da agulha, ou seja, ao ser destrancado, infunde automaticamente.* (P5)

*Tinha que ser na agulha; ao picar-se, ficava aberto. Se é para utilização única, ou seja, ao sistema ser rompido, não se pode voltar a fechá-lo.* (P7)

*Teria que ser um sistema ON/OFF, ou algo do tipo na ponta, e que não precisássemos desses clamps.* (P1)

### Tema 4: Tubuladura/equipo

O equipo do protótipo é acoplado diretamente à bomba elastomérica, e a sua extremidade é conectada ao dispositivo de acesso. Ele mede aproximadamente 1,20 metros e foi considerado grande pelos participantes (Figura 5).

**Figura 5:** Processo da etapa de decisão para o equipo

*A minha experiência com bomba de insulina é que consiga fazer a volta no próprio paciente. Às vezes, ele coloca o dispositivo no tecido subcutâneo desse lado e ele permite que faça a volta nele para ficar do outro lado, ou até no bolso.* (P3)

*Eu acho que é muito grande, se for para estar perto do paciente. Se for para dar a volta ao corpo, ok, mas se não for, vai ficar muito fio solto, e as pessoas têm a probabilidade de apertar e estar a impedir o fluxo, obstruir.* (P6)

Em último aspecto, diante da oportunidade de alcançar pacientes que deambulam, foi proposto que o protótipo semifuncional do DHS tivesse uma bolsa como acessório para colocar a bomba elastomérica dentro dela, assim facilitando carregá-la.

*Ao pensarmos que se a pessoa não está deitada ou está a fazer alguma coisa, tem que andar sempre com isso na mão, e isso não é prático. Tem umas malinhas que o doente põe o aparelho dentro delas e faz a vida dele normal, e pode de fato andar.* (P5)

## DISCUSSÃO

Neste estudo, construímos o primeiro protótipo semifuncional do DHS a partir de dispositivos disponíveis no mercado, e as contribuições do grupo focal heterogêneo foram fundamentais para reunir informações iniciais para aprimorar o *design* desse protótipo e perceber seu potencial de aceitação. A busca da inter-relação entre várias áreas do conhecimento para discutir o mesmo objeto de estudo resultou na produção de novas perspectivas sobre a realidade abordada. Sendo assim, destaca-se a interdisciplinaridade no campo das tecnologias em saúde, uma vez que se alinha ao objetivo de otimizar os meios para o seu desenvolvimento^(14-15)^.

Diante dessa realidade, é essencial ressaltar a decisão de estabelecer o critério de inclusão do primeiro grupo focal, reconhecendo a importância da abordagem técnica para a construção do protótipo. Todavia, na perspectiva de que o seu aprimoramento deve ser progressivo, destacamos a participação dos usuários finais nesse processo como condição sem a qual não se alcançará o sucesso.

É importante destacar o DHS como um facilitador na gestão do tempo dos profissionais de saúde, pois elimina algumas etapas de preparo dos materiais necessários nos ambientes de trabalho. A falta de tempo nos serviços de saúde, ou mesmo na casa do doente, é considerada um fator que aumenta a precarização da assistência e diminui a qualidade do cuidado^(16)^.

Estratégias que permitam melhorar a gestão do tempo possuem um papel importante. Elas impactam diretamente os resultados clínicos^(17)^, a organização e os custos, possibilitando ampliar a capacidade e o acesso aos serviços, principalmente aos dispensados nos hospitais, pois facilitam a transição do cuidado para a atenção primária e para a comunidade, onde alguns procedimentos e cuidados específicos podem ser realizados, como no caso da hidratação^(18)^.

Posto isso, o DHS pode ser considerado aquisição profícua, visto que possui seus componentes em um dispositivo único, de uso simplificado, previamente preenchido, apresentado no modelo de um *kit*, com infusão contínua pré-determinada pelo fabricante.

Entretanto, é importante manter o diálogo com as dimensões econômicas, uma vez que, na incorporação de novas tecnologias em saúde, o valor do produto é um dos fatores a serem analisados^(19)^. O DHS, por ser considerado um *kit* completo, de uso único e pré-preenchido, agrega um maior custo de produção, transporte e aquisição tanto ao consumidor de forma direta quanto aos provedores dos cuidados em saúde, podendo ser uma limitação de interesse ao mercado.

Entretanto, essa hipótese precisa ser avaliada a partir de uma observação analítica que compare a prestação dessa mesma intervenção - hidratação pelo subcutâneo - no ambiente hospitalar, por exemplo. Nesse caso, a inclusão de outras variáveis, como o custo da mão de obra, e dos fatores sociais relacionados ao deslocamento do doente e, por ventura, do seu acompanhante, pode influenciar esses resultados econômicos^(18,20)^. A utilização do DHS no domicílio, por exemplo, pode ser uma medida preventiva e capaz de reduzir uso de urgências e emergências, principalmente na população de idosos frágeis.

Nesse contexto, em alinhamento com os resultados da pesquisa, o ambiente domiciliar foi destacado como possível cenário de uso do DHS. A assistência domiciliar tem se revelado como alternativa à intervenção hospitalar, de caráter complementar a essa, objetivando reduzir a demanda de internações e diminuir o período de hospitalização do doente^(18)^. Além disso, permite que o doente continue no seu contexto familiar e oportuniza uma maior aplicabilidade dos cuidados centrados na pessoa, priorizando um cuidado personalizado e colaborativo^(18,21)^.

Atrelado a esse fato, o envelhecimento populacional ocasiona uma maior busca à assistência domiciliar. Com o aumento da expectativa de vida, nota-se o crescimento dos fatores de risco associados às Doenças Crônicas Não Transmissíveis (DCNT) que, junto ao envelhecimento, geram limitações que podem desenvolver incapacidades funcionais permanentes ou temporárias^(21-24)^. Sendo assim, os cuidados domiciliares tornam-se prioritários, seja na prevenção de agravos, tratamento, reabilitação, ou cuidados paliativos.

Mediante a essa realidade, o público que recebe cuidados domiciliares será o público-alvo principal do DHS. O DHS incorporado a esse público torna-se atrativo ao mercado e aos sistemas de saúde, pois essa modalidade de cuidado apresenta menores custos para a manutenção do paciente e melhores resultados, comparados aos serviços de internação hospitalar^(18,24)^. Do mesmo modo, o uso do DHS em domicílio pode reduzir as visitas dos profissionais e os custos de seus honorários.

Na assistência domiciliar, cabe ressaltar a importância do cuidador familiar, que desempenha um papel relevante na manutenção do doente no domicílio^(25)^. Esse, por sua vez, pode não ter habilidades para exercer tal atividade, o que requer educação em saúde e oferta de instrumentos que possam facilitar a realização dos cuidados de saúde em casa^(26)^, como no caso da hidratação parenteral. Sendo assim, a proposta do DHS para hidratação subcutânea, simples e de fácil uso, pode permitir o manuseio rápido e confortável por pessoa sem habilitações médicas ou de enfermagem especializada, com riscos minimizados.

O uso de tecnologias no âmbito domiciliar, até então de uso hospitalar, acarreta maior chance de risco de erros e eventos adversos^(27)^. Neste estudo, identificamos algumas barreiras que os cuidadores poderiam enfrentar ao utilizar o DHS, como a angulação da agulha do dispositivo de acesso, pois, para realizar a punção, é necessário angular 45º com a pele^(28)^. Somado ao medo por estarem realizando um gesto técnico, os cuidadores podem não ter compreensão da angulação a ser realizada, aumentando o risco de erros. Dessa forma, a angulação idealizada pelos participantes do grupo focal foi a de 90º, pois causaria menos dúvidas acerca do assunto.

Entretanto, tal característica não favorece a aplicação em pacientes em condições de fragilidade intensa, como a caquexia e sarcopenia elevada, por apresentar espessamento do tecido subcutâneo. Nessas condições, protocolos indicam o uso do cateter não agulhado e a inserção do bisel na angulação de 30º com a pele^(29)^. Portanto, na prática, é crucial que o profissional de saúde avalie as condições físicas do paciente, mas também realize a educação em saúde do familiar/cuidador, a fim de garantir o conforto de ambas as partes ao determinar a melhor angulação da agulha do DHS.

A Aliança Mundial de Segurança do Paciente lançou o terceiro desafio global com a temática de medicação segura, apresentando o empoderamento do paciente, familiar/cuidador como um dos seus objetivos^(30)^. Embora a iniciativa tenha o foco na redução de danos referentes à medicação, considera-se que o empoderamento do paciente e do familiar/cuidador deva acontecer em todas as dimensões, complexidades e densidades tecnológicas do cuidado^(31)^.

Nessa perspectiva, este estudo apresenta pontos relativos à preservação da autonomia e à promoção do autocuidado. A autonomia refere-se à participação do paciente na tomada de decisão sobre questões inerentes ao seu cuidado, em respeito à dignidade^(32)^. Por sua vez, o autocuidado relaciona-se a uma função regulatória, em que se permite que as pessoas realizem sozinhas atividades voltadas à preservação da vida, saúde, desenvolvimento e bem-estar^(33)^.

Considerando que os conceitos de autonomia e do autocuidado são complexos, o desenvolvimento de tecnologias promotoras do empoderamento do paciente é desafiador, uma vez que envolve facilidade de uso, componentes adaptáveis a diversos ambientes e características que permitam o seu uso. Debruçando-se nesses conceitos, a apresentação do protótipo semifuncional do DHS como um *kit* de uso simplificado e de fácil inserção, além de associá-lo a uma bolsa acessória, é característica que contribui para a autonomia e para o autocuidado do paciente.

### Limitações do estudo

Foram limitações: um único grupo focal, apesar de tratar-se da primeira fase do estudo; a restrição da escolaridade do nível superior como critério de elegibilidade; a ausência de critérios na caracterização do perfil, como experiência na clínica e no desenvolvimento de produtos, embora a técnica *snowball* tenha direcionado para interessados no campo das tecnologias em saúde; e a não participação de usuários finais (pacientes e familiares). A própria técnica *snowball* também conferiu limitações, por restringir o potencial de abrangência e inclusão do grupo, uma vez que a seleção dos participantes acontece a partir do vínculo entre os mesmos, que podem compartilhar pensamentos e opiniões. O uso de dispositivos disponíveis no mercado também limitou a prototipagem, como no caso do volume da bomba elastomérica, mas, por outro lado, viabilizou economicamente a pesquisa.

### Contribuições para as áreas da enfermagem, saúde, ou políticas públicas

O estudo possui inserção no contexto da tecnologia e inovação, sendo assim, contribui para o crescimento da pesquisa sobre a temática na área da saúde. Contribui para o desenvolvimento de novos dispositivos médicos, especificamente para dar respostas às necessidades do paciente com desidratação leve a moderada no ambiente domiciliar, facilitando a gestão do cuidado em enfermagem e saúde. Tem o compromisso de apresentar aos pesquisadores/profissionais de saúde a importante da prototipagem, que pode ser desenvolvida em várias etapas, devido à necessidade de aprimoramento progressivo.

## CONSIDERAÇÕES FINAIS

Os dispositivos médicos inovadores exercem um papel importante na gestão e promoção da saúde, proporcionando aos profissionais de saúde estratégias para alcançarem uma prestação de cuidado eficiente e segura. Entretanto, esses dispositivos precisam estar em constante aprimoramento para garantir que sua construção e *design* estejam em consonância com os seus usuários finais, e possam proporcionar segurança do tratamento pretendido. Dessa forma, a captação dessas informações sobre o protótipo semifuncional do DHS, por meio do grupo focal, foi positiva e capaz de identificar potencialidades e sugestões para a sua melhoria.

No que diz respeito ao grupo focal, apesar de os resultados obtidos serem satisfatórios, recomenda-se a realização de futuros estudos para o aprimoramento do protótipo final do DHS, com amostras maiores e homogêneas para trazer mais robustez a esse processo. Além disso, focando na usabilidade do DHS, serão necessários estudos que apresentem metodologias envolvendo a testagem do protótipo final em diferentes fases de desenvolvimento do produto.
